# ‘Breaking the stigma’: a qualitative study on how public perceptions affect individuals with Parkinson’s disease – a nurse specialist perspective

**DOI:** 10.1186/s12877-025-06538-9

**Published:** 2025-11-17

**Authors:** Sophie Crooks, Gary Mitchell, Lisa Wynne, Gillian Carter

**Affiliations:** 1 School of Nursing and Midwifery, Queen’s University Belfast, Belfast, Northern Ireland UK; 2 Parkinson’s Ireland, Dublin, Ireland

**Keywords:** Parkinson’s disease, PD, Experiences, Lived experience, Public perception, Stigma, Public, Healthcare, Quality of life, Parkinson’s nurse

## Abstract

**Background:**

Parkinson’s disease (PD) is a chronic neurological disorder that affects around 24,000 people in Ireland. Despite being relatively common, awareness and understanding of the condition remain limited, often leading to misconceptions, stigma, and social isolation for those diagnosed. This study aimed to investigate how these challenges impact individuals with PD, drawing on the experiences and insights of Parkinson’s disease nurse specialists (PDNS). By exploring the perspectives of PDNS, this research seeks to highlight the effects of stigma and public misunderstanding on the quality of life of those living with PD.

**Methods:**

Semi-structured interviews and focus groups were conducted with 10 PDNs working in Northern Ireland and the Republic of Ireland between May and June 2024. The study employed an interpretivist approach and reflexive thematic analysis following Braun and Clarke’s six-step framework. Ethical approval was obtained prior to data collection.

**Results:**

Five main themes were developed from the analysis: (1) Public knowledge of PD and Stigma, (2) Lived Experiences, (3) Support Systems and Services, (4) Increasing awareness of Parkinson’s Disease, and (5) The Role of the PD Nurse. PDNS noted that limited public awareness of PD leads to misconceptions and stigma, negatively impacting the quality of life for those affected. PDNS also emphasised that raising awareness through education, campaigns, and their own role can help improve outcomes for individuals living with PD.

**Discussion:**

The study highlights the complex relationship between public understanding, perceptions, and the quality of life for individuals with PD, as viewed through the lens of Parkinson’s disease nurse specialists. Findings suggest that stigma and a lack of awareness contribute to emotional and social challenges, while strong support networks and public education can significantly enhance the experiences of those living with PD. The results underscore the need for widespread education, not only among the public but also within the healthcare sector, to better support individuals with PD in Ireland. Future research and targeted interventions should focus on increasing societal awareness to improve the lived experiences and well-being of those affected by the condition.

**Supplementary Information:**

The online version contains supplementary material available at 10.1186/s12877-025-06538-9.

## Background

Parkinson’s disease (PD) is a progressive neurodegenerative disorder primarily affecting motor function, with common symptoms including tremors, bradykinesia, rigidity, and postural instability [1]. Non-motor symptoms, such as cognitive impairment, mood disturbances, and autonomic dysfunction, further contribute to the disease burden [1]. While the exact cause of PD remains unclear, a combination of genetic and environmental factors is implicated [2]. The neurological disorder affects over 10 million people worldwide [3] and around 24,000 people across the island of Ireland, and with an aging population, this number is expected to rise [4].

Beyond its clinical manifestations, PD has profound social and psychological consequences for individuals living with the condition [5]. People with PD frequently encounter stigma, misunderstanding, and social isolation, all of which can negatively affect their mental well-being and overall quality of life [6]. Research indicates that public stigma surrounding PD largely stems from a lack of awareness and persistent misconceptions, including the belief that PD exclusively affects older adults or that its symptoms are limited to motor dysfunction [7].

A recent scoping review of 23 studies highlighted widespread gaps in public knowledge regarding PD’s symptoms, causes, and treatment, reinforcing negative societal attitudes that contribute to stigma, isolation, and reduced independence for individuals with the disease [8]. These misconceptions exacerbate challenges in multiple areas, including mental health, social interactions, employment opportunities, and access to appropriate support services [9–12]. Furthermore, the experiences of individuals with PD are shaped not only by their symptoms but also by societal perceptions and interactions [13].

Evidence suggests that social stigma and the public’s lack of understanding can significantly contribute to psychological distress among individuals with PD, increasing the risk of anxiety and depression [10, 14, 15]. A systematic review of 22 studies further identified that PD remains widely misunderstood, with stereotypes surrounding symptomatology, age-related biases, and even supernatural beliefs influencing public attitudes [9]. Additionally, the review found that stigma appears to be driven by factors such as disease duration, symptom severity, and inadequate public education, often exacerbated by misleading media portrayals of the condition [10, 12, 15–21]. Stigma can manifest in various forms, including discrimination and social exclusion, even among healthcare professionals [10, 11, 22]. This stigma not only undermines the mental health of those with PD leading to feelings of shame, embarrassment, and social withdrawal, but also limits their participation in social and professional settings [10, 11, 20, 21, 23, 24]. This can often result in reduced social engagement, heightened emotional distress, and a profound sense of exclusion from the community [10, 11, 14–16, 23, 25–27]. Despite these challenges, many individuals with PD demonstrate resilience, employing coping strategies such as seeking social support, engaging in advocacy, and fostering positive social connections [10, 19, 24]. While stigma presents significant barriers, proactive community education and awareness campaigns have the potential to reshape societal attitudes, reducing misconceptions and improving the overall quality of life for those living with PD [9].

In Ireland specifically, there is a significant gap in research exploring public perceptions of PD and their direct impact on individuals living with the disease. Without a clear understanding of how societal attitudes influence the daily lives of people with PD, efforts to improve awareness, reduce stigma, and enhance support systems remain limited.

Parkinson’s disease nurse specialists (PDNS) play a crucial role in the care and management of individuals living with PD, providing not only medical support but also guidance on symptom management, medication adherence, and overall quality of life [28, 29]. Through their long-term, often home- and community-based relationships with patients, PDNS witness first-hand the daily challenges people with PD face, including the effects of public misconceptions, stigma, and social exclusion [30]. While the lived experiences of people with PD remain central to understanding the social impact of the disease, the perspectives of PDNS provide a distinct and complementary lens. Unlike patients, who speak from individual experience, PDNS observe a wide range of experiences across many individuals and contexts, allowing them to identify common patterns, recurring barriers, and gaps in public awareness. These insights are not simply descriptive; they influence how PDNS deliver care, advocate for their patients, and design education for families and communities.

Understanding how PDNS interpret the lived experience of PD is therefore critical, as their perceptions shape the support strategies, public messaging, and interventions they develop. Examining their perspectives also allows for comparison with patient accounts, helping to identify areas where professional understandings align, or diverge, from those of people with PD, which can inform training and improve care. By exploring PDNS viewpoints, our study does not treat nurses as proxies for people with PD but seeks to understand how their interpretations contribute to the broader ecosystem of care and advocacy. To investigate this, we conducted qualitative interviews and focus groups with PDNS across Ireland and Northern Ireland, exploring their observations of public misconceptions and stigma, and how these insights can inform future awareness campaigns and policy initiatives aimed at fostering a more supportive and inclusive society for those living with PD.

## Methods

### Aim

To explore how public perception and understanding affects people living with PD through the views of PDNS.

### Objectives

To explore the impact of public perception and understanding on individuals living with PD by gathering insights from PDNS in Northern Ireland (NI) and the Republic of Ireland (ROI).

To examine the experiences and perspectives of PDNS regarding the challenges faced by people with PD in their communities.

To investigate how PDNS contribute to raising public awareness and addressing misconceptions about PD within their communities.

### Design

An interpretivist approach was adopted by the researcher due to the exploratory nature of the research. This approach adopts the belief that through interaction with the world around them, individuals assign their own meaning and values to their experiences [31]. Semi-structured interviews and focus groups were conducted with PDNS working in NI and ROI between May and June 2024. This phase of the study was conducted from the perspective of the PDNs to explore how the public knowledge of their disease affects people living with the condition. Interviews with PDNs in ROI took place online and the focus group with PDNS from NI took place in person.

### Sample

The process of recruitment and data collection was guided by purposive sampling via gatekeepers within Parkinson’s Ireland (PI) and Parkinson’s UK NI. PI and Parkinson’s UK NI are charities that offer support services for those living with PD across the UK and Ireland. These charities support approximately 12,000 people living with PD in ROI and around 4,000 people in NI [32, 33]. Only nurses residing on the island of Ireland who specialised in PD and who work with people living with PD were included in this phase of the study.

### Recruitment

PDNS were invited to take part in online or face-to-face interviews exploring how public perception of PD affects those living with the condition. Two gatekeepers from Parkinson’s Ireland and Parkinson’s UK NI supported recruitment, as they had direct links with PDNS and could identify those interested. Study information, including a Participant Invitation Letter, Expression of Interest Form, and Information Sheet, was distributed via email or in hard copy at networking events. Completed forms were returned to the gatekeeper and passed to the researcher, who contacted participants to arrange interviews.

To maximise participation, interviews were scheduled at convenient times. With approximately 17 PDNS working across the island of Ireland, the aim was to engage around eight from each region. Informed consent was obtained before each interview. In-person participants signed printed consent forms, while online participants received digital forms in advance. Consent was reviewed verbally and recorded at the start of each session. All recordings complied with GDPR (Ireland) and the Data Protection Act (2018) (NI). Data was stored securely on a private, password-protected computer.

### Description of sample

One focus group was conducted in NI with 5 specialist PD and movement disorder nurses. All 8 nurses in NI were scheduled to attend the focus group, however, due to a change of circumstances, three were unable to attend. Of the 5 nurses who attended the focus groups, all were female, working as specialist nurses with people with PD across each health and social care trust in NI. Five online semi-structured interviews were conducted with PDNS across ROI. Two were female and the remaining 3 were male. All PDNS were aged between 32 and 55 years old and provided care in a range of settings including hospitals and community. The length of time each PDNS had been in post ranged from 6 years to 16 years. On average, each PDNS supports patients spanning a wide age range, from 40 to 90 years. This highlights the nurses’ extensive experience supporting individuals across all stages of PD.

### Data collection

A schedule of open-ended questions was developed specifically for this study to explore how public knowledge of PD affects those living with the condition and their experiences within the community. The questions focused on, (1) experiences of people with PD; (2) support and services available; and (3) strategies for raising awareness. The same interview schedule was used for both interviews and focus groups. The guide for interviews and focus group can be seen in Supplementary File 1.

Focus groups supported interactive discussion among PDNS, encouraging shared insights not always captured in individual interviews [34]. Semi-structured interviews offered flexibility for PDNS to reflect on their professional experience with PD care. The interview schedule was guided by themes from a prior systematic review [9] and progressed from lived experience to public awareness. Questions were piloted with two independent individuals, resulting in minor wording and order adjustments.

Data collection occurred between May and June 2024 with 10 PDNS. The focus group with NI nurses was held in a familiar hotel setting; 5 of 8 invited participants attended. Those unable to participate were offered alternatives but not followed up, in line with ethical guidelines. ROI interviews were conducted online due to geographical constraints and scheduled at participants’ convenience. Each session lasted approximately 60 min.

### Ethical considerations

Ethical approval was granted by the Faculty of Medicine and Health Sciences, Queen’s University Belfast (MHLS23_132). Written informed consent was obtained, and all procedures adhered to the Declaration of Helsinki. Data were securely stored and accessible only to the research team. Interview transcripts and quotations were anonymised to ensure participant confidentiality.

### Data analysis

The interviews were both video and audio recorded, allowing the researcher to engage fully in discussions without the distraction of taking notes. All recordings were transcribed verbatim to ensure the accuracy of the data. A manual analysis approach was chosen, facilitating a more immersive interaction with the data.

Reflexive thematic analysis was conducted following Braun and Clarke’s six-step framework (2022) [35], which includes: becoming familiar with the data, generating initial codes, identifying potential themes, reviewing themes, defining themes, and producing the final report. Once the initial codes were established, they were reviewed by other team members (GM & GC), who contributed to refining and finalising the themes. A coding example is provided in Supplementary File 2.

### Trustworthiness

To enhance data reliability, this research followed Lincoln and Guba’s (1985) framework, emphasising four key criteria: credibility, transferability, dependability, and confirmability [36]. Participants were actively engaged in the research process and given the opportunity to review both raw data and identified themes, ensuring transparency and inclusivity. Rigorous record-keeping practices were maintained throughout the study. Additionally, regular meetings with the research team were conducted to facilitate in-depth discussions and establish a comprehensive audit trail, reinforcing the integrity of the research approach.

## Results

Thematic analysis identified five key themes, providing insight into PDNS perspectives on the experiences of people with PD within their communities. These themes include: (1) Public knowledge of PD and Stigma, (2) Lived Experiences, (3) Support Systems and Services, (4) Increasing awareness of Parkinson’s Disease, and (5) The Role of the PD Nurse.

### Theme one: public knowledge of PD and stigma

#### Lack of public knowledge

Public understanding of PD remains significantly limited, with widespread misconceptions contributing to stigma and inadequate support. PDNS consistently described public knowledge as poor, emphasising a general lack of awareness about the condition.

“[Public knowledge is] probably poor.” – P7.

“[Public knowledge is] very limited.” – P4.

Beyond this general lack of awareness, PDNS noted that people fail to grasp how complex the disease is.

“I don’t think people understand the complexity of it and the huge amount of symptoms that go along with it.” – P6.

Not only was public knowledge perceived to be poor, a substantial knowledge gap persists particularly among healthcare professionals. According to PDNS, many hospital staff demonstrate a limited understanding of the multifaceted nature of PD.

“Unfortunately, even in hospitals, you see people looking after patients who don’t have a very good understanding of what Parkinson’s is, especially the non-motor side of it.” – P6.

While motor symptoms such as tremors are widely recognised, non-motor symptoms which are often more challenging to manage, receive disproportionately less attention in both clinical and public discourse, according to PDNS.

“’I don’t have a tremor, so I can’t have Parkinson’s.’ It’s not understood that, you know, it’s only one of many symptoms.” – P9.

#### Misconceptions and stigma

Beyond a general lack of awareness, PDNS note that misconceptions about PD contribute to stigma and misunderstanding about the disease.

One common misconception highlighted during the study is that PD only affects older adults, with little awareness that younger individuals can also be diagnosed.

*“It’s very much considered an old person’s disease.” – P6*.

*“The usual perception is that it’s an older person’s condition when it’s not.” – P9*.

This stereotype often portrays PD as a condition associated with frailty, stooped posture, and slow, shuffling movement.

*“Stooped-over old person*,* yeah. They don’t see it as a younger person’s condition.” – P4*.

*“[People associate PD with] a stooped old man shuffling up and down the street with a stick.” – P8*.

PDNS also highlighted the misconception that PD is the same as, or closely linked to, other neurological disorders, particularly dementia. This leads to the false belief that everyone with PD will inevitably experience cognitive decline.

*“Years ago*,* people used to ask*,* ‘Is that the same as Alzheimer’s?’ There’s always been this misconception that all brain diseases are the same and that people with Parkinson’s are always demented or have cognitive impairment.” – P8*.

*“There are a lot of misconceptions about the dementia aspect of PD.” – P8*.

According to PDNS, another widespread belief is that tremors are a universal symptom. This creates confusion when people with PD do not exhibit tremors, leading to misunderstanding about their diagnosis.

*“When people think of Parkinson’s*,* they just think of tremors.” – P7*.

*“A lot of people think tremor is the main feature*,* but some people don’t have tremors at all.” – P1*.

PDNS explained that some believe PD results in inevitable and rapid decline, leading to assumptions about wheelchairs and early death.

*“People assume Parkinson’s means immobility…that everyone will end up in a wheelchair.” – P8*.

*“Most people think it’s a death sentence when it’s not.” – P9*.

*“They assume that within a certain timeframe*,* they’ll become wheelchair-bound. They think*,* ‘Oh*,* this is the end.’” – P10*.

Some PDNS shared that patients are sometimes mistaken as being drunk due to speech or movement difficulties.

*“Many people have presumed they were drunk because their speech might be a little lower or slurred or if they’re tripping and falling.” – P8*.

*“People thought they’re drunk.” – P1*.

*“[A patient] was leaving work*,* got into their car*,* and drove down the road. They were stopped by the police because of a member of the public had phoned to say that they were under the influence of alcohol.” – P3*.

Misconceptions extend into the healthcare system. PDNS explain how lack of understanding can lead to stigma, especially around medication timing, with patients unfairly seen as difficult by other healthcare professionals.

*“There is definitely a lack of understanding*,* and that stigma that patients are impatient or difficult*,* especially when it comes to medication management.” – P6*.

PDNS pointed to limited media attention as a reason stigma persists. Other conditions often dominate headlines due to new treatments and trials.

*“There have been other conditions that have been talked about and spoke about and that are brought to media more so than Parkinson’s.” – P2*.

#### Impact of stigma and misconceptions

PDNS in this study highlighted how stigma and misconceptions impact those living with the disease, including the psychological and social consequences for those affected. The PDNS perceived that a lack of understanding can leave individuals living with PD feeling unheard and misunderstood, negatively impacting their well-being and sense of support.

*“[It impacts patients] negatively because they don’t feel heard or understood.” – P6*.

*“It can take more of an emotional toll on people.” – P6*.

PDNS explain how the lack of awareness among healthcare professionals further exacerbates these challenges, stating patients may begin to question themselves, feeling as though they are being difficult or a burden, and even apologise for needing their medication.

*“They might start questioning themselves*,* maybe I am being difficult*,* or nearly making them feel as though they need to apologise for wanting their medications.” – P6*.

PDNS describe how the stigma and misconceptions experienced by those with PD often leads to significant social withdrawal. The PDNS reported that many individuals with PD avoid social situations for fear of being judged or misunderstood. Also, the concern of being watched or misinterpreted often leads to self-imposed social exclusion and often worsens their symptoms.

*“They’re aware somebody is looking at their tremor*,* which makes their tremor worse because their anxiety increases. A lot of people would move away from social situations.” – P9*.

*“They are conscious about being looked at by others due to motor symptoms. So they are socially withdrawn mostly because of these reasons.” – P10*.

PDNS expressed how many individuals with PD choose to hide their diagnosis due to fear of being treated differently or unfairly. The stigma associated with the condition often leads them to keep their diagnosis private to avoid judgement or altered perceptions from others.

*“They don’t tend to tell people because they feel they’re going to be treated differently.” – P9*.

PDNS also highlighted how stigma impacts individuals with PD in the workplace. While employers are generally accommodating, some individuals still choose to conceal their condition due to embarrassment or fear of discrimination. This stigma can affect their professional identity and confidence, according to PDNS. However, legislative protections help ensure workplace support and accommodations.

*“I still have one person who hides their condition from their employer. They’re embarrassed to say anything in case they’re treated differently.” – P9*.

*“Employers*,* in general*,* are usually quite good…but there is an onus on them from a legislative perspective to provide accommodations*,* and that helps.” – P9*.

This theme highlights the widespread lack of awareness about PD among both the public and healthcare professionals and its impact on those living with the condition, through the lens of a PDNS. It underscores how stigma and misconceptions, as perceived by PDNS, contribute to challenges such as depression, anxiety, and social isolation, further affecting the well-being and quality of life of individuals with PD.

### Theme two: lived experiences

The theme of ‘lived experiences’ explores how individuals with PD navigate life within their communities, as seen from the perspective of PDNS. It highlights both positive and negative experiences, while also acknowledging variations influenced by factors such as age and gender.

#### Positive experiences

In this theme, PDNS highlighted the positive aspects of living with a PD diagnosis from the perspective of those living with the disease. In particular, PDNS highlighted the strong support systems that people often experience within their communities.

*“As it [Parkinson’s] was advancing*,* they all worked together… she became the centre of the community.” – P9*.

This sense of support also extends to the workplace. PDNS explain that while some people with PD initially experience fear and uncertainty about how their diagnosis will impact their careers, many find reassurance in a supportive work environment.

*“A few of my early-onset patients feel very supported at work…Initially*,* they were so nervous*,* thinking*,* ‘Oh my God*,* I have this diagnosis*,* am I going to lose my job*,* my mortgage? How am I going to cope?’ But they’ve been well supported.” – P9*.

It was emphasised by PDNS how community support also plays a crucial role in fostering a Parkinson’s-friendly environment. PDNS explain how a collective effort from family, friends, and the broader community can make a meaningful difference in the lives of those living with the condition.

*“They use the phrase*,* ‘It takes a village to raise a child.’ It also takes a village to care for older people and those with chronic conditions.” – P9*.

*“This particular area is much more Parkinson’s-friendly.” – P9*.

#### Negative experiences

Despite there being many positive experiences, PDNS also highlighted several negative experiences faced by individuals with PD, particularly encounters with impatience from the public. The PDNS felt that many individuals with PD experience frustration from others due to the slower movements associated with their condition, while some have even been asked to stop their involuntary tremors.

*“I’ve seen one of my patients with Parkinson’s who works here in the hospital… Her movement was quite slow*,* and no one else knew that she had Parkinson’s*,* but I did. I could see the impatience of a man watching her*,* thinking*,* ‘Oh god*,* would you just come on and put in the file?’ I saw him looking at his watch.” – P6*.

*“I’ve seen a couple of times where a person has asked a patient to stop shaking”– P7*.

While family members are often a key source of support, their lack of understanding can sometimes negatively impact individuals with PD. PDNS explain how in some cases, the reactions of others inadvertently hinder a person’s ability to manage their condition, leading to unnecessary panic or exaggeration of challenges that the individual may not have initially perceived as significant.

*“What we see a lot is that family members can sometimes negatively impact the process of someone getting on with their life.” – P7*.

*“Even though the patient might be okay*,* the family starts panicking. Then other people get involved*,* and things become catastrophised. Suddenly*,* everything feels like a much bigger deal than it actually was for the person themselves.” – P7*.

#### Differences in experiences

PDNS highlighted differences in how individuals with PD cope with their condition, as well as how they are perceived and treated by the public. Stigma and societal reactions varied, with some individuals facing more challenges than others.

One significant factor influencing the experience of a PD diagnosis was age. PDNS explain how younger individuals often face greater emotional challenges, as the diagnosis can disrupt their stage of life more profoundly. Concerns such as childcare, pregnancy, menstrual cycle impact, and medication management have been raised. In contrast, older individuals, particularly those in their 80–90 s, were perceived to be more accepting of the condition, as understood by PDNS.

*“But I do think age plays a part because it can be more impactful on people’s lives.” – P6*.

*“It’s definitely more of a con to get Parkinson’s in your youth.” – P7*.

*“I think sometimes older people may be a little bit more accepting.” – P6*.

To better support younger people with PD, one participant emphasised the importance of structuring clinics in a way that groups patients of similar ages and disease stages together. The PDNS believe this approach prevents younger patients from feeling overwhelmed or discouraged by seeing individuals in more advanced stages, such as those using wheelchairs or walkers.

*“When I schedule clinics*,* I try to keep stages together. So if I have people coming in their 40s*,* I’ll make sure there’s another younger person there. I know it can really impact people to come in and see a waiting room filled with wheelchairs*,* walkers*,* and frames… People think*,* ‘Oh*,* this is me now.’” – P6*.

*“There’s one man… He drives a motorcycle*,* comes in wearing his leathers and helmet*,* and I think it’s a great reminder Parkinson’s isn’t just the wheelchair.” – P6*.

This theme highlights the diverse experiences of individuals with PD, shaped by both positive and negative interactions within their communities through the lens of the PDNS. It also emphasises how factors such as age can influence these experiences, underscoring the need for more personalised support and awareness to improve quality of life.

### Theme three: support systems and services

The theme of ‘Support Systems and Services’ explores the importance of accessible support for individuals with PD from the perspective of PDNS. It examines the services currently available, highlights examples of support models from other conditions that could be adapted for PD, and discusses the barriers individuals face in accessing care, revealing gaps in existing support systems.

#### Importance of support

Support groups and community-based services were seen as playing a vital role in enhancing the well-being of individuals with PD, according to PDNS. They highlighted the benefits of engaging with structured services such as support groups and exercise classes. Those who accessed these resources reported improved outcomes and a greater sense of well-being.

One PDNS emphasised the strong correlation between engagement in these initiatives and positive disease management for those with PD.

*“People who go to support groups or exercise classes or try new things… they’re the ones who do really*,* really well.” – P7*.

PDNS highlight that local support groups were frequently cited as one of the most valuable resources available. These groups provide not only a space for shared experiences and peer support but also access to educational and therapeutic services.

*“The best resource people have*,* I think*,* is local support groups. When that’s in place in the community*,* they often have a group of people who can share experiences*,* and they get their own speakers*,* so there’s more education available to them.” – P7*.

A recurring theme among PDNS was the alleviation of isolation through shared experiences. As stated by PDNS, many individuals with PD struggle with feelings of loneliness, and engaging with a group of peers can provide significant emotional relief. One participant described the profound impact of isolation on those with PD.

*“There’s nothing more isolating or*,* I suppose*,* lonely than feeling like you’re the only person with something and that someone else isn’t… not having a shared experience. That’s really hard.” – P6*.

The importance of community in managing PD was also emphasised by PDNS. The presence of others who share similar experiences fosters a sense of security and emotional resilience for those living with the disease.

*“That sense of community is really important for people. Everyone needs someone going through similar experiences to them.” – P6*.

PDNS note beyond emotional well-being, this communal support system also provides practical benefits where people with PD can exchange strategies for managing symptoms and navigating daily challenges.

*“They’re able to discuss their problem then and there*,* and they get tips*,* take tips from other people. It’s not them alone who are fighting with this condition.” – P10*.

#### Types of support and services available

PDNS highlighted a diverse range of support services available to individuals with PD, including community groups, exercise programmes, and charitable organisations. Initiatives such as the ‘Men’s Shed’ were seen as beneficial, offering structured environments for social engagement through activities like woodworking, games, and outings.

Another example mentioned was the ‘Making Memories Café’ for dementia patients, which organises visits to museums and landmarks. PDNS suggested a similar approach could benefit those with PD.

*“We’ve set up a Making Memories Café for dementia patients. Even if we were to set up a Making Movement Café or something like that from a Parkinson’s point of view*,* it would be great.” – P8*.

Charitable organisations, particularly Parkinson’s Ireland, were viewed as crucial sources of information, advocacy, and local support.

*“We have a lot of good charities and organisations that are there for people. So*,* whether it’s support groups*,* exercise classes*,* or different types of groups like that*,* there are a lot of great initiatives out there if people are able to find them.” – P7*.

PDNS also emphasised the positive impact of exercise-based programmes, which offer both physical and emotional benefits. Programmes like Move for Parkinson’s, Tai Chi, and football were noted as supportive, alongside community groups such as the GAA.

*“I think if there were communities in GAA clubs all around*,* it would make a big difference to people.” – P6*.

One PDNS highlighted the benefits of Rocksteady Boxing in fostering well-being and connection.

*“They feel better attending services like Rocksteady Boxing or any future sessions. When they talk about things*,* they feel like they have more support.” – P10*.

These findings reflect the PDNS view that structured activities promote both health and social connection for people with PD.

#### Barriers to accessing support

Despite the availability of support groups and community services, several barriers prevent individuals with PD from fully engaging. PDNS identified challenges such as mobility limitations, anxiety, stigma, and personal preferences that affect participation.

A common obstacle cited by PDNS was anxiety and mobility issues, which can limit a person’s ability to leave home and access support.

*“It comes down to how good and confident they feel at getting out.” – P8*.

Age influenced experiences with support groups. Younger individuals often felt out of place in groups dominated by older people, which could discourage attendance.

*“A younger person with Parkinson’s isn’t going to get the same out of a support group as someone in their 60s. There’s the stigma of being the only young person there*,* surrounded by older people*,* and that can be quite difficult.” – P7*.

Stigma and fear of being labelled were additional barriers. PDNS note some people with PD avoided services to keep their diagnosis private.

*“Some patients don’t want to be noticed or labelled as having Parkinson’s.” – P4*.

PDNS also noted that accepting the diagnosis was difficult for some.

*“They have to try to accept it themselves before they even talk about it to others.” – P2*.

#### Gaps in services and support

A significant gap in services for individuals with PD was identified, particularly concerning access to specialised healthcare professionals. PDNS highlighted the urgent need for increased availability of speech and language therapy (SLT), physiotherapy, and occupational therapy (OT). One PDNS emphasised the disparity in services for younger individuals.

*“There’s a big shortage for people under 65.”*- P9.

Similarly, access to PD specialist nurses was deemed insufficient by PDNS themselves. Comparisons were drawn to stroke services, where multiple nurses are available per trust, whereas PD services often operate with only one or two nurses, making comprehensive care difficult.

*“This is most of the Parkinson’s nurses in Northern Ireland here and there’s not very many of us. Whereas my previous history was a stroke nurse*,* there’s a stroke nurse in every area*,* I mean the trust would have had*,* I mean*,* six or so per trust whereas we have one or two*,* per trust for Parkinson’s.” – P1*.

PDNS also repeatedly stressed the need for enhanced psychological and counselling services for individuals with PD, alongside more awareness.

*“More awareness of this condition and more psychological support definitely makes such a difference.”* – P4.

Another key concern for PDNS was the absence of Parkinson’s-specific counselling services, which are available for conditions like multiple sclerosis (MS).

*“I think the MS society has a counselling service…we don’t have anything like that for people with Parkinson’s.”* – P3.

Access to mental health services was further restricted by long waiting lists. In some cases, PDNS explain that individuals with PD were even denied referrals to memory assessment services due to their diagnosis, an issue one PDNS described as discriminatory.

*“The waiting lists are so long*,* and actually*,* Parkinson’s can be a barrier to accessing mental health services. We asked for referrals to the Memory Assessment service*,* and they were declined on the basis that they have Parkinson’s. I mean*,* that’s discrimination.”* – P1.

To address these gaps, PDNS advocated for the establishment of a dedicated multidisciplinary PD team, consisting of speech therapists, physiotherapists, and occupational therapists, similar to existing stroke services.

*“When I worked in stroke*,* we had a stroke-specific speech therapist*,* physio*,* OT*,* a whole team. There’s no team for Parkinson’s” – P1*.

The shortage of PD-specific consultants was another pressing concern for PDNS, with calls for additional specialist roles to improve patient care.

*“I know consultants are very difficult for Parkinson’s and so to get an actual consultant with an interest in Parkinson’s*,* that definitely is a massive thing at the moment.” – P2*.

For individuals unable to attend clinics, greater community-based support was recommended. PDNS highlighted the importance of facilitating at-home care to ensure ongoing access to essential services.

*“Having that community support from healthcare teams*,* you know*,* that’s all they have….MDT*,* speech and language things like that*,* and it’s all about facilitating people staying at home.”* – P3.

However, a recurrent theme was the lack of funding for these initiatives.

*“No funding or support [is] going into the services.”* – P3.

This theme highlights the critical role of support systems in the lives of individuals with Parkinson’s disease, as observed by PDNS. While existing services provide valuable assistance, gaps and barriers remain, limiting access to essential care. Adapting successful support models from other conditions could help bridge these gaps and improve overall support for people with PD.

### Theme four: increasing awareness of parkinson’s disease

The theme of ‘Increasing Awareness of Parkinson’s Disease’ examines the importance of educating the public about PD from the perspective of PDNS. It explores various strategies for raising awareness, including public campaigns and educational initiatives, to enhance understanding and support for those living with the condition.

#### Awareness campaigns

A key strategy mentioned by PDNS in the interviews for improving public understanding of PD is increasing its visibility in mainstream media. PDNS highlighted the importance of featuring more individuals with PD on television to challenge misconceptions and encourage open discussions.

*“[It is a] good thing having more people on TV with PD.”*- P6.

One impactful example shared by one PDNS was a woman with PD who appeared on the news after collaborating on an innovative walker that allowed her to run. Her story sparked meaningful conversations among her family and colleagues, helping those unfamiliar with PD gain a deeper understanding.

*“It definitely broke a few*,* I suppose*,* stigmas.”* – P6.

Personal narratives were recognised as one of the most effective ways to raise awareness by PDNS. Hearing firsthand experiences were seen to help humanise the condition and shift public perception.

*“I think people telling their stories is very powerful*,* and I think that has a big impact on people.”* – P6.

PDNS mentioned high-profile individuals who have openly spoken about their PD were also seen as influential advocates. Figures such as broadcaster Jeremy Paxman and Irish journalist Anne Marie O’Connor were praised for bringing attention to the condition.

*“More high-profile people who have done stuff like Jeremy Paxman and Anne Marie*,* the Irish journalist—you know*,* I think that’s really positive.”* – P6.

Additionally, the idea of appointing a well-known public figure with PD as an ambassador was suggested to further boost awareness.

*“An ambassador for PD*,* like somebody who has it*,* TV soaps with characters with Parkinson’s*,* more media representation.”* – P1.

Social media was also identified as a powerful tool for reaching the public, particularly younger generations who engage more with digital platforms than traditional media.

*“Social media maybe… should be used a bit more because it’s so… people nearly watch it more than TV now*,* don’t they?”* – P6.

Large-scale public awareness campaigns were seen as essential in changing how PD is perceived in society. PDNS pointed to the success of awareness initiatives for conditions like ADHD and autism and suggested a similar approach could benefit PD.

*“I do think of ADHD… people are more aware of people with ADHD or autism… they’re more aware of it and know how to work around it.”* – P9.

One example discussed was Parkinson’s Ireland’s campaign on the 40 symptoms of PD. While informative, some PDNS felt that listing symptoms such as hallucinations and cognitive impairment could be overwhelming or even frightening to those unfamiliar with the condition.

*“Maybe that would be frightening for people.”* – P8.

PDNS also emphasised the importance of maintaining a positive tone in awareness campaigns, rather than focusing solely on the negative aspects of the disease.

*“I think just keeping that positive slant on advertisements or campaigns*,* they work. The positivity works rather than*,* you know*,* negativity in those campaigns.”* – P8.

Some PDNS suggested incorporating humour and entertainment to engage the public. Specifically, comedy was seen as an effective tool for leaving a lasting impact.

*“Something done through a comedy slant*,* I feel*,* works because if people laugh*,* it sparks an emotion and they’ll remember that happy emotion… a comedy sketch.”* – P8.

#### Education

One of the key factors of the reduced public awareness of PD is the lack of education, not only among the general public but also within the healthcare sector. PDNS touched on the importance of equipping medical professionals, caregivers, and families with the necessary knowledge to support individuals with PD effectively.

*“Education on the medical professional or healthcare professional for the patients*,* the families*,* and the carers*,* just educate them.”* – P8.

Increasing discussions around PD in both professional and social settings was seen as essential for improving awareness by PDNS. Simple but effective measures such as displaying informative materials in waiting rooms or increasing PD-related coverage in national newspapers were suggested as ways to reach a wider audience.

*“What we try to do is have lots of like physical flyers in the waiting room or hanging up. That’s usually the best way that we can get people to be aware of something.”* – P7.

Another practical step involved making informational leaflets more widely available in public spaces such as GP clinics, libraries, and community centres.

*“More education in public areas and more availability of leaflets for them wherever they go.”* – P10.

PDNS also recommended increasing charity involvement in fundraising and awareness events. Holding events in accessible locations such as nursing homes, libraries, and integrated care facilities was seen as a valuable way to reach senior citizens and other key groups.

*“They are raising more awareness by conducting more fundraising… linking with all the nursing homes and linking with all the libraries and GP services and with integrated care support.” – P5*.

#### Importance of increased awareness

PDNS emphasised how increasing public knowledge about PD could help challenge stereotypes and reduce discomfort in social interactions.

*“I think people would react better. I think when people know more about Parkinson’s*,* they won’t have those stereotypes and they’ll be more patient. They’ll also just have more understanding for people and not come at it with discomfort or negativity. It can only be positive*,* really.”* – P1.

Awareness was also linked to greater patience and empathy. By educating people on PD symptoms, individuals with PD may receive more acceptance and practical support in daily life.

*“Help them to have a better understanding of the symptoms and maybe if they do come in contact with somebody with Parkinson’s*,* more acceptance and patience.”* – P3.

Beyond social acceptance, raising awareness was seen as crucial for increasing financial and governmental support for PD services. PDNS noted that public understanding often leads to better funding and access to healthcare resources.

*“What comes with more awareness comes more money from government*,* and more money means more resources.”* – P7.

A comparison was made between PD and MND, highlighting disparities in public awareness and funding. Despite PD being more common, it does not receive the same level of resources as rarer conditions.

*“In the Republic of Ireland for sure*,* there’s huge public awareness about conditions like motor neurone disease*,* and they have huge access to resources. They’ve got really good funding in public healthcare*,* and the motor neuron disease society has four full-time nurses. That’s a really rare condition*,* whereas Parkinson’s isn’t so rare*,* yet the services just aren’t even comparable.”* – P7.

High-profile individuals were recognised by PDNS as key advocates in raising awareness and shifting public perceptions of PD. Celebrities who openly discuss their experiences can help bring attention to the condition.

*“Michael J. Fox being a big name for PD - I think people like him are hugely beneficial.”* – P7.

This theme highlights the importance of increasing awareness of PD, as suggested by PDNS. They identified various ways to achieve this, including awareness campaigns, informational leaflets, greater media representation, education in schools, and increased involvement from charities. Raising awareness through these efforts can help challenge stigmas, bringing about acceptance and support within communities.

### Theme five: the role of the PD nurse

The theme of ‘The Role of the PD Nurse’ highlights the vital role that PDNS play in patient care. Beyond providing medical support, PDNS develop a deep understanding of each individual, offering emotional support, educating both patients and their families, and advocating for their needs. Their holistic approach helps improve the quality of life for those living with PD.

PDNS mentioned the crucial role that they play in both patient care and raising awareness.

*“I think definitely there is a place for us because there’s such understanding for people with*,* you know*,* from nurses who are specialising in that area that it’s nearly it’s it’s nearly wasteful or something.”* – P6.

Beyond medical care, PDNS often act as emotional support figures for patients and their families. Their ability to listen, reassure, and provide comfort was seen as an essential part of their role.

*“Sometimes it feels very much like counselling.”* – P6.

*“But counselling and listening and reassuring and being with someone*,* you know*,* if they’re upset even*,* or*,* you know*,* and that can happen on the phone*,* but it can happen in person too.”* – P6.

PDNS also play a key role in educating both patients and medical staff. One participant highlighted that even among nurses and doctors, awareness of PD remains limited.

*“Even with graduate like nurses and doctors*,* their knowledge of Parkinson’s is usually like fairly poor.”* – P7.

To bridge this gap, PDNS frequently speak at support groups and hospitals, ensuring that both professionals and the public have access to accurate information.

Another major responsibility of PDNS is connecting newly diagnosed patients with resources in their community. This support can lead to increased engagement with exercise classes, support groups, and other beneficial activities, sometimes reducing the need for medication.

*“So if someone is diagnosed and then I meet them about four weeks later for a post-diagnostic session*,* talk to them about stuff and then point them in directions of what is available to them in their community. And then in a good situation*,* the person kind of takes things on*,* reads a little bit and goes out and joins a club or a society or an exercise class.”* – P7.

PDNS were also described as vital links between healthcare professionals and the PD community. One participant stressed the importance of their leadership role in ensuring best practices are followed.

*“I think it’s really important as our role is to be leaders for other healthcare professionals and student nurses*,* say*,* or student doctors that they see the way things can be done right and how effective that is when it’s done right.”* – P8.

Education remains a core part of their work, particularly in highlighting key issues such as medication timing and speech difficulties to ensure patients receive the best possible care.

The findings from interviews and focus groups with PDNS highlight the gap in public knowledge about PD, as well as the persistent misconceptions and stigma surrounding the condition. These misunderstandings can lead to social isolation, a lack of empathy, and barriers to accessing appropriate support. However, PDNS emphasised that education and awareness initiatives, both for the general public and healthcare professionals, are key to overcoming these challenges. PDNS play a crucial role not only in providing specialist care and emotional support to individuals with PD, their families, and caregivers but also in advocating for greater awareness and understanding within society. By enhancing support services, improving public education, and ensuring the visibility of PD in mainstream conversations, PDNS can help promote a more informed and compassionate community, ultimately improving the quality of life for those living with the condition.

## Discussion

This study highlights significant gaps in public knowledge about PD contributing to stigma, misconceptions, and inadequate support, as perceived by PDNS, however, these challenges are not unique to PD. Similar patterns of misunderstanding and social exclusion have been documented in research on other neurological and chronic conditions in Ireland. Studies on multiple sclerosis (MS) and epilepsy, for example, reveal persistent public misconceptions, such as the assumption that these conditions primarily affect older adults or inevitably lead to cognitive decline [37, 38]. As with PD, individuals with MS frequently report feeling misunderstood, particularly when experiencing invisible symptoms like fatigue or cognitive impairment [39].

One of the main themes found was the stigma attached to people living with PD and the impact this can have, not only on their mental health and well-being but also on their likelihood to socialise in public. The impact of stigma on social participation and well-being is a recurring theme across chronic conditions. According to PDNS, individuals with PD often experience social withdrawal due to fear of judgement, a pattern that mirrors findings from research on mental health conditions like depression and anxiety, where stigma is strongly linked to isolation and reduced willingness to seek help [40, 41]. This issue is reflected internationally, with a systematic review [9] highlighting widespread stigma associated with PD and its negative impact on those living with the condition.

Additionally, employment concerns raised by PD patients reflect broader issues of workplace discrimination in chronic illness research, emphasising the need for stronger legal protections and greater employer awareness [42–44].

A key finding of this study is the critical role of support systems in enhancing the well-being of individuals with PD. Consistent with previous research, structured support groups, exercise programs, and community-based initiatives play a vital role in disease management and emotional resilience [45, 46]. Similar to MS and MND, PD patients benefit significantly from peer support, which reduces isolation and facilitates coping strategies [47–49]. However, unlike MND, which receives substantial public healthcare funding and has a dedicated network of specialist nurses, PD services in Ireland remain underfunded and fragmented.

Barriers to accessing support including mobility limitations, stigma, and geographic constraints were also prominent findings in this study. These challenges are consistent with prior research indicating that individuals with neurological conditions in Ireland, particularly those in rural areas, often struggle to access appropriate healthcare services [50]. These findings also reflect international trends, where research from Canada shows people with PD living in rural areas often face reduced access to support and services [51].

The findings briefly highlight how PDNS reflect on their professional identity, particularly in relation to other healthcare professionals and their advocacy roles. Participants noted a lack of understanding about PD among non-specialist staff, prompting PDNS to take on educator and advocate roles, both for patients and within clinical teams. These dynamics raise broader questions about how condition-specific professionals are positioned and valued, especially in comparison to colleagues in better-resourced specialties, such as stroke.

Such reflections point to wider hierarchies in healthcare, where the visibility and funding of a condition often shape the authority and recognition of those working in it [52]. PDNS compared their services unfavourably to those in stroke care, despite similar needs for multidisciplinary input. This resonates with challenges faced by professionals supporting other long-term conditions like dementia, where expertise is essential but often underacknowledged [53]. Elevating these roles could strengthen interdisciplinary care and improve outcomes across chronic disease settings.

Finally, public awareness and education are pivotal in reshaping societal perceptions of PD. While conditions such as breast cancer, with the success of the ‘Wear it Pink’ campaign [54], and MND, through initiatives like the ALS Association Ice Bucket Challenge campaign [55], have benefited from widespread and impactful awareness efforts, PD remains comparatively underrepresented. PDNS in this study also emphasised the importance of media representation, ambassador-led initiatives, and targeted educational programs to challenge misconceptions, strategies that have been effective in other neurological conditions such as Stephen Hawking and Rob Burrows bringing significant awareness to MND.

These campaigns and initiatives are crucial for increasing public awareness of PD, creating more supportive and inclusive communities for those affected. Greater awareness can also draw attention from policymakers and funding bodies, potentially leading to improved access to services and the development of additional support resources. Ultimately, these efforts aim to enhance the quality of life for individuals living with PD, ensuring they receive the care and support they need.

### Strengths and limitations

The strength of this paper was that it offered a platform for PDNS to share insights into the experiences of individuals living with PD across the island of Ireland. These nurses, who work closely with patients, offer a unique perspective on the challenges and realities faced by those affected by the disease. While the paper highlights public perceptions of PD as understood by specialist nurses in Ireland, it is important to note that these perspectives may not fully reflect the views and experiences of people living with PD themselves, nor those from other regions of the world.

Despite efforts to engage all PDNS across the island of Ireland, time constraints and demanding work schedules prevented full participation from all eligible nurses. As a result, the findings of this study are based solely on the perspectives of those who were able to participate, and may not fully represent the views of those who were unable to take part.

## Conclusion

This study highlights the significant impact PD has on individuals’ daily lives, particularly in navigating their life in the community and managing challenges from the perspective of PDNS. PDNS frequently stressed the importance of support networks, including family, employers, and the wider community, in ensuring resilience and promoting inclusion. However, public stigma and misconceptions surrounding PD further complicate these experiences, reinforcing the need for greater awareness and education for the public and healthcare professionals. Creating a more informed and supportive society is essential in improving the quality of life and overall well-being of those living with PD.

## Supplementary Information

Supplementary Material 1.

Supplementary Material 2.

## Data Availability

All data generated or analysed during this study are included in this published article and its supplementary information files.

## Abbreviations

PD: Parkinson’s Disease
PDN: Parkinson’s Disease Nurse
QoL: Quality of Life
PI: Parkinson’s Ireland
NI: Northern Ireland
ROI: Republic of Ireland
MS: Multiple Sclerosis
MND: Motor Neurone Disease
