# Supplementary material for: ‘Breaking the stigma’: a qualitative study on how public perceptions affect individuals with Parkinson’s disease – a nurse specialist perspective
Source: BMC Geriatr. 2025 Nov 17;25:910. doi: 10.1186/s12877-025-06538-9 (PMC12625472; doi:10.1186/s12877-025-06538-9)
Supplement: Supplementary file 1 — Supplementary Material 1. [file 12877_2025_6538_MOESM1_ESM.docx]

**Introduction:**

1. Can you briefly describe your role as a specialist Parkinson's disease nurse and your interactions with people living with Parkinson's in the community?

**Understanding Experiences:**

1. From your perspective, what are the key challenges or experiences that individuals with Parkinson's face while living in their communities?
2. Can you share specific examples of situations where people with Parkinson's encounter difficulties or unique experiences within their communities?

**Support and Services:**

1. What kinds of support and services do you think people with Parkinson's need to enhance their quality of life in the community?
2. In your experience, are there any gaps or areas where improvements could be made in terms of community-based support for individuals with Parkinson's?

**Communication and Awareness:**

1. How can communication between healthcare professionals, including yourself, and the community contribute to a better understanding of Parkinson's challenges?
2. What role can PD nurses play in raising awareness about Parkinson's disease within local communities?

**Social and Psychological Impact:**

1. How does Parkinson's impact the social and psychological well-being of individuals living in the community, and what can be done to address these aspects?
2. Are there ways you've seen the community respond positively to individuals with Parkinson's, and how has that affected their experiences?

**Barriers to Engagement:**

1. Are there factors that might prevent people with Parkinson's from actively participating in community activities or accessing services? How can these barriers be overcome?

**Collaboration and Partnerships:**

1. How do you collaborate with other professionals, community organizations, or local authorities to improve the experiences of people with Parkinson's in their communities?
2. Are there successful examples of partnerships that have positively impacted individuals with Parkinson's?

**Future Improvements:**

1. In your view, what steps could be taken to create more inclusive and supportive communities for people with Parkinson's?
2. What changes or initiatives do you think would have the most significant impact on improving the lives of individuals with Parkinson's in their local areas?

**Personal Reflection:**

1. Have you had any particularly memorable experiences or stories from your work that highlight the importance of community support for people with Parkinson's?
2. Is there anything else you would like to share about your role and your perspective on the experiences of people with Parkinson's in their communities?
