# Supplementary material for: ‘Breaking the stigma’: a qualitative study on how public perceptions affect individuals with Parkinson’s disease – a nurse specialist perspective
Source: BMC Geriatr. 2025 Nov 17;25:910. doi: 10.1186/s12877-025-06538-9 (PMC12625472; doi:10.1186/s12877-025-06538-9)
Supplement: Supplementary file 2 — Supplementary Material 2. [file 12877_2025_6538_MOESM2_ESM.docx]

1. Codes were generated for each individual interview and narrowed down into subthemes. Similar themes were combined and narrowed down to 13 which were colour coded:

| Positive experiences | Misconceptions and stigma | Types of support and services available | Importance of increased awareness |
| --- | --- | --- | --- |
| Lack of public knowledge | Negative experiences | Differences in experiences | Impact of stigma and misconceptions |
| Awareness campaigns | Importance of support | Education | Barriers to accessing support |
| The role of the PD nurse | Gaps in services and support |  |  |

1. Themes were combined and reduced further to 5 and relevant subthemes were generated from this:

| **Theme One: Public Knowledge of PD and Stigma** | **Theme Two: Lived Experiences** | **Theme Three: Support Systems and Services** | **Theme Four: Increasing Awareness of Parkinson’s Disease** | **Theme Five: The role of the PD nurse** |
| --- | --- | --- | --- | --- |
| Lack of public knowledge | Positive experiences | Importance of Support | Awareness campaigns | The Role of the PD nurse |
| Misconceptions and stigma | Negative experiences | Types of support and services available | Education |  |
| Impact of stigma and misconceptions | Differences in experiences | Barriers to accessing support | Importance of increased awareness |  |
|  |  | Gaps in services and support |  |  |
|  |  |  |  |  |

**One theme example:**

| **Theme** | **Subtheme** | **Code** | **Description** | **Example Quotations** |
| --- | --- | --- | --- | --- |
| Public Knowledge of PD and Stigma | Lack of public knowledge | General lack of public knowledge of PD | Discusses the general lack of awareness both the public and healthcare professionals have of PD in Ireland. | *"[Public knowledge is] very limited." – RM* |
|  |  | Lack of knowledge of healthcare professionals |  | *"Unfortunately, even in hospitals, you see people looking after patients who don’t have a very good understanding of what Parkinson's is, especially the non-motor side of it." – AM* |
|  |  | Unaware of complexity of PD |  | *"I don't think people understand the complexity of it and the huge amount of symptoms that go along with it." – AM* |
|  | Misconceptions and stigma | PD as an ‘Old Person’s Disease’ | Explores the commons misconceptions and stigmas the public have associated with PD | *“It’s very much considered an old person’s disease.” – AM* |
|  |  | Confusion between PD and other neurological disorders |  |  |
|  |  | Assumptions about rapid and severe disease progression |  | *“Years ago, people used to ask, ‘Is that the same as Alzheimer’s?’ There’s always been this misconception that all brain diseases are the same and that people with Parkinson’s are always demented or have cognitive impairment.” – BM* |
|  |  | Stigma related to motor symptoms e.g. under the influence of alcohol |  |  |
|  |  | Misconceptions and stigma from healthcare professionals |  |  |
|  |  | Reasons for misconceptions and stigma in PD |  |  |
|  |  |  |  | *“When people think of Parkinson’s, they just think of tremors.” – BD* |
|  |  |  |  | *“People assume Parkinson’s means immobility…that everyone will end up in a wheelchair.” – BM* |
|  |  |  |  | *“People thought they’re drunk.” – SH* |
|  |  |  |  | *“There is definitely a lack of understanding, and that stigma that patients are impatient or difficult, especially when it comes to medication management.” – AM* |
|  |  |  |  | *“There have been other conditions that have been talked about and spoke about and that do be brought to media more so than Parkinson’s.” – FM* |
|  | Impact of Stigma and Misconceptions | Psychological and emotional toll | How public stigma and misconceptions impact those living with the disease | *“It can take more of an emotional toll on people.” – AM* |
|  |  | Healthcare related challenges |  |  |
|  |  | Social withdrawal and isolation |  | *“I’m sad, really, to think that they might start questioning themselves, maybe I am being difficult, or nearly making them feel as though they need to apologise for wanting their medications.” – AM* |
|  |  | Hiding the diagnosis |  |  |
|  |  | Workplace stigma |  | *“They are conscious about being looked at by others due to motor symptoms. So they are socially withdrawn mostly because of these reasons.” – RM* |
|  |  |  |  | *“They don’t tend to tell people because they feel they’re going to be treated differently.” – GD* |
|  |  |  |  | *“I still have one person who hides their condition from their employer. They’re embarrassed to say anything in case they’re treated differently. They’re a young man, and they don’t want to be identified as ‘that person’ because they’re usually very high-flying and successful.” – GD* |
